# Purifying selection during maternal inheritance of mammalian mtDNA depends on autophagy and bottleneck size

**DOI:** 10.1080/15548627.2026.2650772

**Published:** 2026-03-31

**Authors:** Polyxeni Papadea, Nils-Göran Larsson

**Affiliations:** Department of Medical Biochemistry and Biophysics, Division of Molecular Metabolism, Karolinska Institutet, Stockholm, Sweden

**Keywords:** Bottleneck, maternal transmission, mitochondria, mitophagy, mtDNA mutations

## Abstract

Mammalian mitochondrial DNA (mtDNA) is transmitted asexually without recombination and accumulates mutations at a high rate, which eventually should cause a mutational meltdown. Two processes operating in the maternal germline, the genetic bottleneck and purifying selection, are counteracting this decline but the exact molecular mechanisms and their possible link remain incompletely understood. To address this, we investigated the role of autophagy and mtDNA copy number in shaping purifying selection during maternal mtDNA transmission. Using a carefully designed breeding strategy in mice expressing a proofreading-deficient mitochondrial DNA polymerase, we generated animals carrying random mtDNA mutations and simultaneously introduced moderately decreased or increased mtDNA copy number, or impaired autophagy. Mutation patterns in control animals closely resembled those observed in humans, showing strong purifying selection against non-synonymous mutations, particularly in oxidative phosphorylation (OXPHOS) genes. Our recent work provides new insight by identifying autophagy as a key mediator of germline purifying selection of mtDNA. Moreover, we demonstrate that mtDNA copy number directly influences the efficiency of purifying selection, revealing that these two processes are functionally interconnected.

Maternal inheritance of mtDNA presents an evolutionary paradox due to its high mutation rate and the lack of germline recombination. The famous geneticist Hermann Muller predicted that such asexual genome transmission inevitably would lead to mutational meltdown (*Muller’s ratchet*). Two mechanisms in the mammalian maternal germline prevent this outcome: the genetic bottleneck, which stochastically transmits only a subset of maternal mtDNA variants, and purifying selection, an active process eliminating pathogenic mutations. In human pedigrees, mutated and wild-type mtDNA frequently coexist (heteroplasmy), and the genetic bottleneck typically generates wide variation in mutation levels among siblings. Children with exclusively mutated mtDNA (homoplasmy) are rarely born, reflecting the presence of strong purifying selection. Consistently, large-scale mtDNA sequencing studies reveal robust purifying selection, particularly against nonsynonymous mutations in the 13 mtDNA-encoded subunits of the OXPHOS system. Together, the genetic bottleneck and purifying selection play critical roles in shaping mtDNA inheritance and human mitochondrial disease. However, the underlying mechanisms and the extent to which these processes are mechanistically linked have remained unclear.

In our study [1], we used an elaborate breeding scheme of mutant mice to introduce a large set of random mtDNA mutations and studied maternal transmission when mtDNA copy number was altered or autophagy was impaired. Mice with random mtDNA mutations were generated by creating female hemizygous mtDNA mutator mice (*Polg*^−/mut^), which are functionally equivalent to mtDNA mutator (*Polg*^mut/mut^) mice as they only express a proof-reading-deficient version of the mitochondrial DNA polymerase (Figure 1(A)). The *Polg*^−/mut^ mice were generated by mating heterozygous knockout females (*Polg*^+/-^) and heterozygous mtDNA mutator males (*Polg*^+/mut^) mice. The mtDNA mutator allele was thus introduced from males to ensure that *Polg*^−/mut^ females (F0 generation) have not inherited mtDNA mutations from their mothers, which otherwise would have been a confounding factor for the study. Thereafter, hemizygous females were bred for three generations to remove the *Polg*^mut^ allele and to obtain mice with high levels of random mtDNA mutations in the N3 generation (Figure 1(A)). We combined this breeding scheme with alleles that alter the expression of TFAM (transcription factor A, mitochondrial) to moderately decrease (*Tfam*^*+/-*^) or increase (*Tfam*^*+/OE*^) mtDNA copy number (Figure 1(B)). We also introduced knockout alleles for *Ulk2* (unc-51 like autophagy activating kinase 2; *ulk2*^−/−^) and *Bcl2l13* (BCL2 like 13; *bcl2l13*^−/−^) to decrease general and receptor-mediated mitophagy during mtDNA transmission (Figure 1(C)).

**Figure 1.**
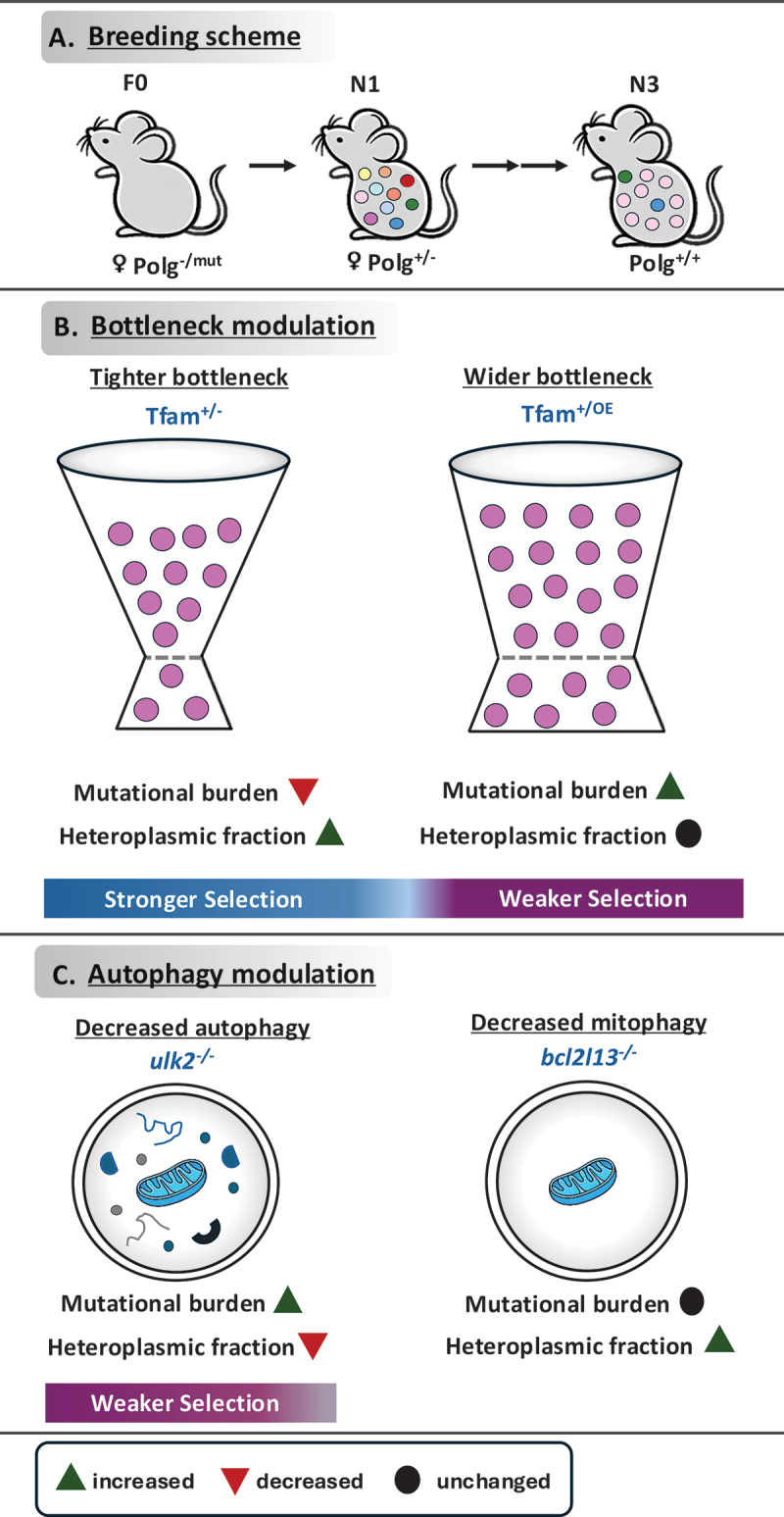
Effect of bottleneck and autophagy modulation on the transmission of mtDNA mutations. (A) Breeding strategy to obtain mice that contain randomly generated mtDNA mutations. (B) The impact of manipulation of the bottleneck size on the heteroplasmic fraction and mutational burden of mtDNA. A tighter bottleneck leads to stronger purifying selection whereas weaker selection is observed when the bottleneck is wider. (C) The effect of impaired general autophagy (*ulk2*^−/−^) and mitophagy (*bcl2l13*^−/−^) on the heteroplasmic fraction and mutational burden. Decreased general autophagy leads to weaker purifying selection.

Using high-depth Illumina sequencing, 3366 unique variants at 3176 positions were identified across the 161 mice analyzed from the different experimental groups. The detected variants were distributed along all genes of mouse mtDNA, enabling an unbiased genome evaluation. The generated mutation pattern in the control group was very similar to the purifying selection pattern observed in humans and revealed more synonymous than non-synonymous variants in protein-coding genes. Mimicking the human situation, the gene encoding MT-CO1 (mitochondrially encoded cytochrome c oxidase I) was subjected to strong purifying selection whereas the selection against mutations in the genes encoding ATP synthase subunits (*mt-Atp6* and *mt-Atp8*) was more relaxed.

Next, we assessed the heteroplasmic fraction (HF, proportion of mutant:total mtDNA) and the number of variants (the mutational burden) of maternally transmitted mtDNA mutations in the N3 generation of the different experimental groups (*Tfam*^*+/-*^; *Tfam*^*+/OE*^; *ulk2*^−/−^ or *bcl2l13*^−/−^) in comparison with the control group. Surprisingly, we found that the *Tfam*^*+/-*^ group has an increased median HF and a reduced burden of transmitted mutations. In contrast, the *Tfam*^*+/OE*^ group has an increased mutational burden. These results show that mtDNA copy number directly affects purifying selection (Figure 1(B)). When general autophagy was decreased in the *ulk2*^−/−^ group, we found a decreased HF and an increased mutational burden of transmitted mutations, showing that autophagy is important for purifying selection of mtDNA in the maternal germ line. In contrast, loss of BCL2L13, which is involved in receptor-mediated autophagy of mitochondria (mitophagy), results in an increased HF. The likely explanation is that BCL2L13 removes certain types of mtDNA mutations present at high levels, which explains why *bcl2l13*^−/−^ mice have offspring with a wider distribution of heteroplasmy levels but display a minimal impact on total mutation levels.

In conclusion, the published study provides genetic evidence that the bottleneck and purifying selection by autophagy are functionally linked in the maternal germline and are essential for maintaining mtDNA genome integrity over generations. A tighter bottleneck caused by mtDNA copy number reduction will result in a higher HF that exposes the variants to stronger selection, eventually leading to the transmission of less variants to the offspring. Conversely, impairment of general autophagy by ULK2 depletion, will lead to weaker selection eventually causing more variants to be transmitted to the offspring at low heteroplasmy levels. Together, these findings provide new insights into the mechanisms governing the transmission of mtDNA variants across generations, which is particularly important for the study of mitochondrial diseases and reproductive interventions. Furthermore, both processes may also contribute to the mitochondrial dysfunction during mammalian aging because somatic mutagenesis during mtDNA replication in postnatal life will have more serious consequences if there are preexisting maternally transmitted mutations.

## Data Availability

There are no datasets associated with this manuscript.
